# Electrocatalytic CO_2_ Reduction Coupled with Water Oxidation by bi- and Tetranuclear Copper Complexes Based on di-2-pyridyl Ketone Ligand

**DOI:** 10.3390/molecules30071544

**Published:** 2025-03-31

**Authors:** Siyuan Yang, Tian Liu, Wenbo Huang, Chengwen Zhang, Mei Wang

**Affiliations:** School of Materials Science and Engineering, Institute for New Energy Materials & Low Carbon Technologies, Tianjin University of Technology, Tianjin 300384, China; yangsiyuan129@stud.tjut.edu.cn (S.Y.); lt18670859926@163.com (T.L.); 19942665525@163.com (W.H.); koakt057@163.com (C.Z.)

**Keywords:** copper complex, dpk ligand, water oxidation, CO_2_ reduction, homogeneous electrocatalysis, bifunctional catalysts

## Abstract

In the field of sustainable energy conversion and storage technologies, copper-based complexes have become a research hotspot due to their efficient and stable catalytic performance. The development of bifunctional catalysts that can simplify catalytic steps, enhance efficiency, and reduce catalyst usage has become an important research area. In this study, we successfully synthesized two copper complexes with different geometries utilizing di(2-pyridyl) ketone as the ligand, [Cu^II^_2_L_2_Cl_2_]·0.5H_2_O (**1**) and [Cu_4_^II^L_4_(OCH_3_)_2_](NO_3_)_2_ (**2**) (L = deprotonated methoxy-di-pyridin-2-yl-methanol), which can serve as homogeneous electrocatalysts for water oxidation and CO_2_ reduction simultaneously. The turnover frequency (TOF) of complexes **1** and **2** for electrocatalytic water oxidation are 7.23 s^−1^ and 0.31 s^−1^ under almost neutral condition (pH = 8.22), respectively. Meanwhile, the TOF of complexes **1** and **2** for the catalytic reduction of CO_2_ to CO are 4.27 s^−1^ and 8.9 s^−1^, respectively. In addition, both complexes remain essentially unchanged during the electrocatalytic water oxidation and electrocatalytic CO_2_ reduction processes, demonstrating good stability. Structural analysis reveals that the distinct catalytic efficiencies originate from their geometric configurations: the binuclear structure of complex **1** facilitates proton-coupled electron transfer during water oxidation, whereas the tetranuclear architecture of complex **2** enhances CO_2_ activation. Complexes **1** and **2** represent the first two copper molecular electrocatalysts capable of catalyzing both water oxidation and CO_2_ reduction. The findings in this work can open up new avenues for the advancement of artificial photosynthesis simulation and the development of bifunctional catalysts for water oxidation and CO_2_ reduction.

## 1. Introduction

In classic artificial photosynthesis systems, the two half-reactions of water oxidation and CO_2_ reduction are of great significance for mimicking natural photosynthesis and achieving sustainable energy conversion [1,2,3,4,5,6]. The protons and electrons generated during the water oxidation reaction are indispensable “raw materials” for the reduction of CO_2_. Electrocatalytic water oxidation, as a source of inexpensive electrons, is a very promising approach for converting freely available energy into chemicals; however, it also faces bottlenecks in energy conversion [7,8,9]. A series of metal complexes have been reported as electrocatalytic water oxidation catalysts with low overpotential and high Faraday efficiency [10,11,12,13,14,15,16]. Meanwhile, CO_2_ is the primary greenhouse gas, and an increase in its concentration in the atmosphere will exacerbate the greenhouse effect, leading to global warming. Against the backdrop of global warming, reducing CO_2_ emissions, achieving carbon neutrality, and peaking carbon emissions have become crucial actions for humanity’s self-rescue [17,18,19]. Therefore, from a societal perspective, achieving sustainable development largely depends on the large-scale utilization of CO_2_ for the production of value-added chemicals [20,21,22,23,24,25,26,27,28,29]. Employing well-defined complexes in homogeneous catalytic reactions not only boosts catalytic efficiency substantially but also enables a molecular-level insight into the catalytic mechanisms of these complexes, ultimately paving the way for the advanced development of related catalysts [30].

**Scheme 1:**
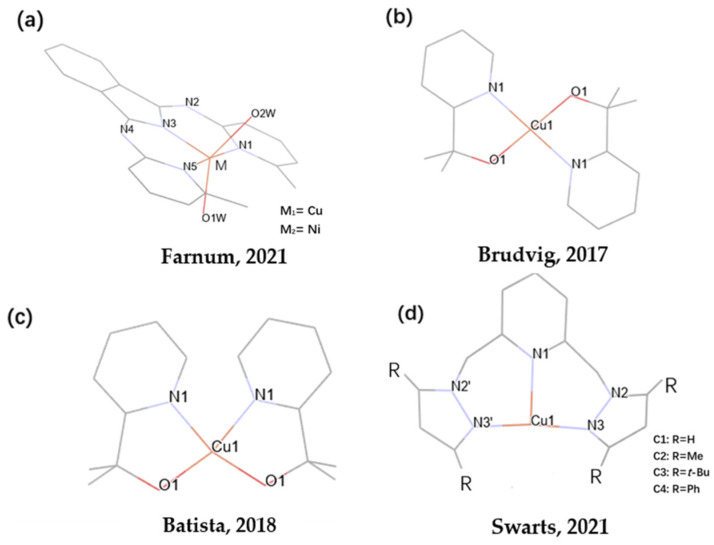
The structures of the complexes (**a**) [Cu^II^(BPI_Me_)(H_2_O)_2_]^+^ and [Ni^II^(BPI_Me_)(H_2_O)_2_]^+^ [35]; (**b**) [Cu^II^(pyalk)_2_] [36]; (**c**) [*cis*-Cu^II^(pyalk)_2_] [37]; (**d**) [Cu^I^L]^+^ (L = bis(pyrazol-1-ylmethyl)pyridine, C1–C4) [38].

A series of metal complexes have been developed as bifunctional electrocatalysts for CO_2_ reduction and water oxidation, such as Pt, Ir, Ru, and Au [31,32,33,34]. Although noble metal complexes exhibit excellent catalytic performance in the fields of electrocatalytic CO_2_ reduction and water oxidation, their large-scale application is somewhat limited due to the scarcity of noble metal reserves and their high prices. Copper-based complexes possess diverse coordination modes and can form stable complexes with various ligands, thereby satisfying the different requirements of water oxidation and CO_2_ reduction reactions. Byron’s team reported two Cu(II) and Ni(II) pincer complexes coordinated by BPI_Me_^−^ [BPI_Me_^−^ = 1,3-bis((6-methylpyridin-2-yl)imino)isoindolin-2-ide] (Scheme 1a), both of which are capable of the electrocatalytic reduction of CO_2_ to CO. However, the copper complex [Cu-BPI_Me_]^+^ exhibited higher catalytic activity for CO_2_ reduction than that of [Ni-BPI_Me_]^+^ [35]. Brudvig’s group reported that the Cu-based water oxidation catalyst [Cu(pyalk)_2_] (pyalk = 2-pyridyl-2-propanoate) (Scheme 1b) exhibited a turnover frequency (TOF, TOF indicates how many reactant molecules each active site of the catalyst can catalyze to convert into products per second or per hour) of approximately 0.7 s^−1^ under alkaline conditions due to the oxidation resistance and strong donor character of the ligand pyalk [36]. Benjamin’s group synthesized the [*cis*-Cu^II^(pyalk)_2_] complex(Scheme 1c), which has been identified as a highly active and robust electrocatalyst for water oxidation under alkaline conditions, displaying a TOF of 0.7 s^−1^ at a pH = 12.5 [37]. In recent years, Swarts’ group has reported a series of novel bis(pyrazol-1-ylmethyl) pyridine-ligated Cu complexes with different electron-donating groups on the pyrazole ring (H, Me, t-Bu, Ph) (Scheme 1d), all of which have excellent water oxidation properties [38]. However, there are still very few reports regarding Cu-based complexes that can be used as bifunctional catalysts for both CO_2_ reduction and water oxidation [39].

Di-2-pyridyl ketone (dpk), which can exhibit multiple coordination modes and chelate through multiple donor sites such as *N*, *N*, *N*, *O* or *N*, *N*, *O* donor atoms, is a versatile bidentate or tridentate ligand [40,41]. The ketone C=O group is prone to nucleophilic addition when it is coordinated to a transition metal center [42]. As a promising candidate for the preparation of a variety of metal complexes, the dpk ligand has three potential donor groups, two 2-pyridyl nitrogens and the carbonyl oxygen, which has aroused the interest of many researchers [43]. The dpk ligand is able to exhibit enhanced back-bonding capacity, and the electrons involved are formally located in suitably oriented t_2g_ orbitals when bound by the two N-atoms to a metal ion or an atom [44]. Considering the redox-active properties for potentials of copper complexes containing *N*-heterocyclic ligands, polydentate ligands such as di(2-pyridyl) ketone (dpk) are crucial components in the preparation of coordination compounds [42,45,46]. McKenzie’s group reported a *fac*-[Re(dpk)(CO)_3_Cl] complex synthesized with a dpk ligand, which can electrocatalyze CO_2_ reduction to CO, because the ligands can help stabilize the reaction intermediates and reduce the activation energy of the reaction [47]. Meanwhile, Bakir et al. prepared and studied an analogous manganese complex coordinated with the dpk ligand, namely *fac*-[Mn(CO)_3_(dpk)Br], which also demonstrated that the dpk ligand played important role in the process of electrocatalytic CO_2_ reduction reactions [48]. Ding’s group reported two complexes with dpk·OH as the ligand, [Co^III^(dpk·OH)_2_]Cl and [Cu_8_(dpk·OH)_8_(OAc)_4_](ClO_4_)_4_, both of which displayed great catalytic performance towards water oxidation owing to the regulation of the dpk·OH ligand [49,50]. Nevertheless, there have been scare reports on bifunctional catalysts synthesized using the dpk ligand that possess both electrocatalytic water oxidation and CO_2_ reduction capabilities, and the catalytic performance of such catalysts urgently needs improvement.

Based on the aforementioned research background, this study utilized di(2-pyridyl) ketone (dpk) as the coordination ligand, ultimately achieving the targeted synthesis of two copper complexes, [Cu^II^_2_L_2_Cl_2_]·0.5H_2_O (**1**) and [Cu_4_^II^L_4_(OCH_3_)_2_](NO_3_)_2_ (**2**) (L = deprotonated methoxy-di-pyridin-2-yl-methanol), and studied the homogeneously electrochemical property of the two complexes. The binuclear complex **1** is composed of two Cu(II) centers with identical coordination environment containing two dpk ligands and two chloride atoms, with each copper atom being pentacoordinated. The tetranuclear complex **2** is coordinated by four dpk ligands and two methoxy groups, wherein two of the copper ions are four-coordinated, and the other two copper ions are six-coordinated. The investigation shows that both complexes **1** and **2** exhibit great electrocatalytic performance for water oxidation and CO_2_ reduction simultaneously. The TOF for the electrocatalytic water oxidation of complexes **1** and **2** are 7.23 s^−1^ and 0.31 s^−1^, respectively. In addition, the TOF for the electrocatalytic reduction of CO_2_ to CO of complexes **1** and **2** are 4.27 s^−1^ and 8.9 s^−1^, respectively. Further, the two complexes developed in this study demonstrate great structural robustness in both CO_2_ reduction and water oxidation tests. This work makes a substantial contribution to expanding the catalytic applications of copper-based complexes and offers an essential guideline for the development of novel catalysts for energy conversion.

## 2. Results and Discussion

### 2.1. dpk·MeOH Synthesis

**Scheme 2:**
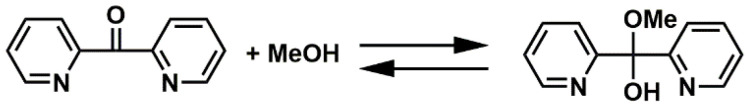
The formation of the dpk·MeOH ligand.

The possibilities for the ligand di(2-pyridyl) ketone (dpk) coordination are substantial, as the ligand can coordinate in either protonated or deprotonated form [44,51]. In the presence of alcohols, (e.g., methanol) the ketone carbonyl can undergo an “alcoholated” (dpk·MeOH) hemiketal derivative (Scheme 2), the formation of which was confirmed by ¹H NMR spectroscopy (Figure S1). The ketone carbonyl can form a gem-diol (dpk·H_2_O) in the presence of water [42,52]. In addition, the dpk ligand can serve as a redox-active ligand to assist in catalytic reactions [53]. We aimed to find the most suitable copper salt, solvent, and reaction temperature to synthesize different kinds of copper complexes with the dpk ligand for electrocatalytic CO_2_ reduction and water oxidation catalysis.

Eventually, by using CuCl_2_·2H_2_O, Cu(NO_3_)_2_·6H_2_O as the salt sources and methanol as the solvent, we successfully isolated a binuclear and a tetranuclear Cu^II^ complex with an “alcoholated” dpk·MeOH. The negatively charged oxygen atom from the deprotonated hydroxy group can increase the likelihood of bridging between metal centers [42].

### 2.2. Analysis of Crystal Structures

**Figure 1:**
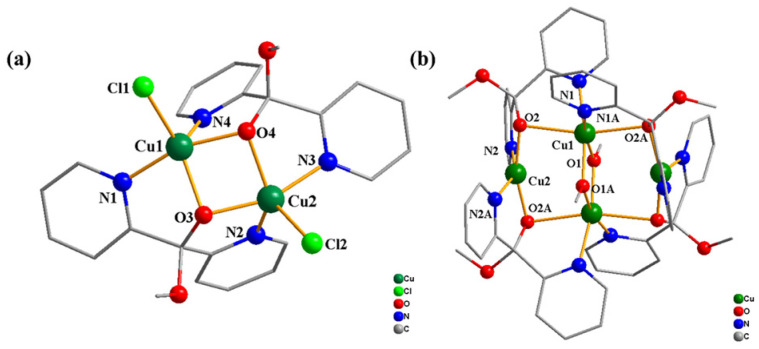
Crystal structures of complexes **1** (**a**) and **2** (**b**), the hydrogen atoms have been omitted for clarity. Color code: Cu: green, O: red, N: blue, Cl: bright green, C: gray.

**Figure 2:**
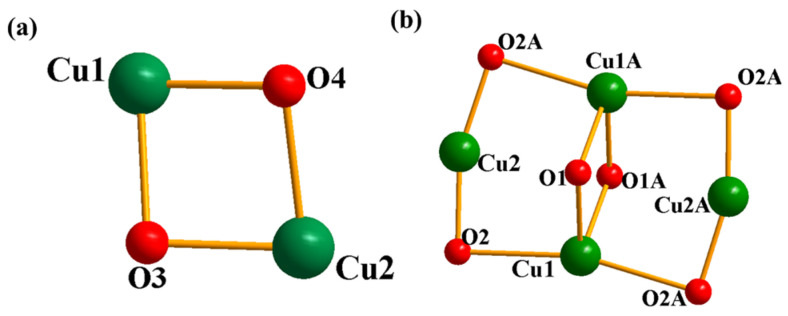
The inorganic core of complexes **1** (**a**) and **2** (**b**).

The X-ray diffraction analysis of single crystals reveals that complex **1** forms crystals in the monoclinic system, belonging to the space group *P*_21_/n, whereas complex **2** crystallizes in the tetragonal system, characterized by the centrosymmetric space group *I*_41_/acd (as shown in Table S1). The structure of complexes **1** and **2** are shown in Figure 1 and Figure 2, while the selected bond angles are listed in Table S2. The binuclear complex **1** is composed of two Cu(II) centers with an identical coordination environment containing two deprotonated dpk·MeOH ligands and two chloride atoms, with each copper atom being pentacoordinated by two N atoms, two O atoms and one Cl atom. The tetranuclear complex **2** is coordinated by four deprotonated dpk·MeOH ligands and two methoxy groups, wherein two of the identical copper ions are four-coordinated by two N and two O atoms, and the other two copper ions are six-coordinated by two O and four N atoms. Electrospray ionization mass spectrometry (ESI-MS) analysis (Figure S2) confirmed that the structures of complexes **1** and **2** are consistent with their proposed compositions.

### 2.3. Electrochemical Properties of Complexes ***1*** and ***2***

**Figure 3:**
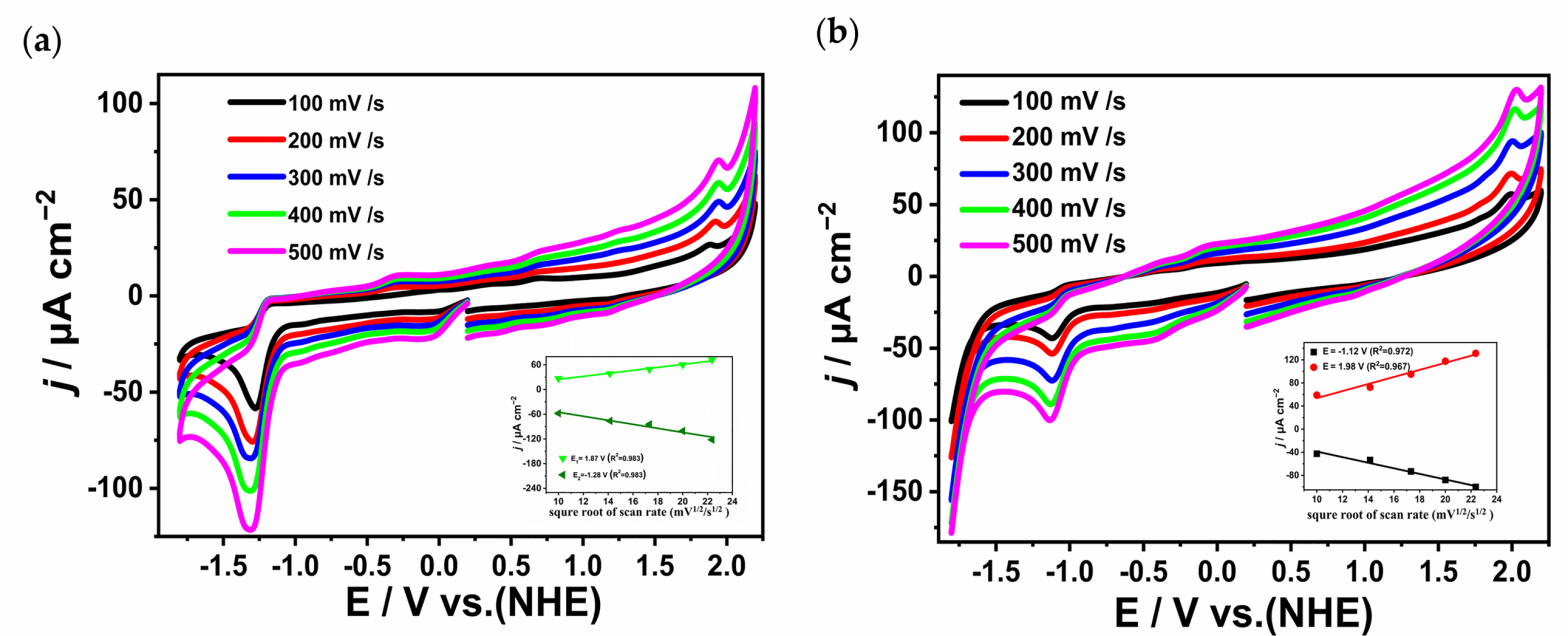
Cyclic voltammetry of 0.2 mmol/L complexes **1** (**a**) and **2** (**b**) in MeCN under 1 atm Ar at scan rate ranging from 100 to 500 mV/s, respectively. (Inset) The linear plots of peak currents versus the square root of the scan rate of complexes **1** and **2**.

To investigate the electrochemical properties of complexes **1** and **2**, cyclic voltammetry tests were conducted in a three-electrode system (all potentials versus NHE). As shown in Figure 3, the CV curves obtained at different scan rates ranging from 100 to 500 mV/s reveal that complex **1** had four irreversible oxidation peaks at −1.17, −0.37, 0.7, and 1.87 V during the anode scan, while two irreversible reduction peaks emerged at −0.02 and −1.28 V under cathodic polarization. For complex **2**, only one oxidation peak at 1.98 V and one reduction peak at −1.12 V were observed. The additional small shoulder peaks in the CV curves of complex **1** compared to those of complex **2** could be attributed to the passivation of the electrode. The waves at −1.28 V and 1.87 V for complex **1** could be assigned to Cu_2_^II^/Cu^I^Cu^II^ and Cu^I^Cu^II^/Cu_2_^II^, respectively. Meanwhile, the peaks at −1.12 V and 1.98 V for complex **2** could be assigned to Cu_4_^II^/Cu^I^Cu_3_^II^ and Cu^I^Cu_3_^II^/Cu_4_^II^, respectively. As shown in Figure S3, we also evaluated these results at 100 mV/s for 10 cycles for both complexes **1** and **2.** In addition, as shown in the inset of Figure 3, as the scan rate varies from 100 to 500 mV/s, the square root of the scan rate (*v*^1/2^) demonstrates a linear dependency with its corresponding peak current (*i_p_*). This provides strong evidence that the redox processes of complexes **1** and **2** are governed by diffusion-controlled mechanisms rather than surface adsorption effects. This behavior is particularly noteworthy for homogeneous electrocatalysts, as it confirms the structural integrity of the complexes during redox cycling and excludes the phenomenon of ligand dissociation.

### 2.4. Study on Electrocatalytic Performance of Water Oxidation of Complexes ***1*** and ***2***

**Figure 4:**
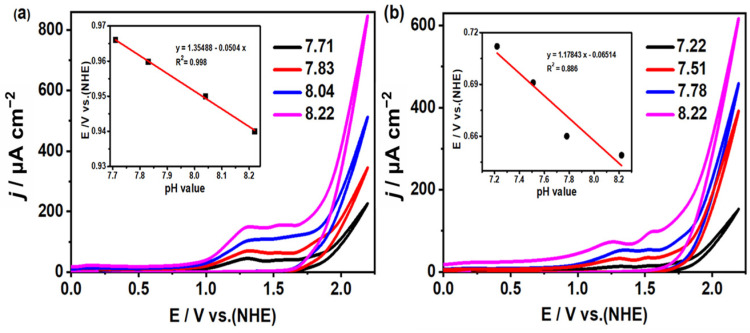
Electrochemical characterization of copper complexes: cyclic voltammetry (CV) profiles of (**a**) complex **1** and (**b**) complex **2** (0.2 mM) in a sodium acetate–acetic acid (NaAc-HAc) buffer system (0.1 M) recorded at a scan rate of 100 mV s^−1^ under Ar-saturated conditions. (Inset) The correlation between the starting voltage and pH value for complexes **1** and **2**.

To investigate the electrocatalytic water oxidation activities of complexes **1** and **2**, cyclic voltammograms of both complexes were measured in buffer solutions with varying pH values. As depicted in Figure 4, under conditions of pH 8.22, complexes **1** and **2** exhibited irreversible oxidation current peaks at potentials around 1.3 V and 1.55 V, respectively. In addition, many oxygen bubbles produced at this point were observed on the surface of GC electrode, which were detected by an Ocean Optics NeoFox-GT oxygen sensor. All this evidence shows that the two complexes can catalyze water oxidation at 1.3 V for complex **1** and 1.55 V for complex **2**, respectively. The onset potentials for the water oxidation of both complexes exhibited pH dependence, with pH response gradients of 50 and 65 mV pH^−1^ for complexes **1** and **2**, respectively (inset of Figure 4). These observations suggest the involvement of a proton-coupled electron transfer (PCET) mechanism. Kinetic studies further revealed a proportional correlation between the catalyst concentration and peak current response (Figure S4), supporting first-order kinetics governing the rate-determining step in the water oxidation process for both complexes.

Using the half-peak potential data obtained from cyclic voltammetry (CV), specifically referencing *E_real_* (O_2_/H_2_O) in Equation (2), we determined that the overpotential (*η*) required for electrocatalytic water oxidation of complex **1** is approximately 400 mV within a pH range of 7.71 to 8.22. Correspondingly, for complex **2**, the overpotential (*η*) needed for the same reaction falls between 690 and 741 mV within a pH range of 7.22 to 8.22. These overpotential values were calculated based on Equations (1) and (2).

(1) $$E_{theooy}\left(O_{2}/H_{2}O\right)=1.23-0.059pH$$

(2) $$\eta =E_{real}\left(O_{2}/H_{2}O\right)-E_{theory}(O_{2}/H_{2}O)$$

Utilizing the reaction kinetic equation (Equation (3)), we can approximately estimate the catalytic turnover frequency (TOF) of the complex under optimal conditions [54,55].

(3) $$\frac{i_{cat}}{i_{p}}=1.424\sqrt{\frac{k_{cat}}{v}}$$

**Figure 5:**
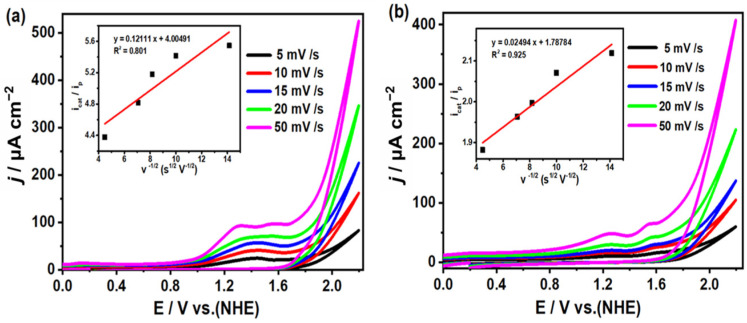
Cyclic voltammograms of complexes **1** (**a**) and **2** (**b**) in 0.1 M acetate buffer under Ar-saturated conditions with a glassy carbon electrode. The inset shows linear fitting plot of *i*_cat_/*i*_p_ and *v*^−1/2^ for TOF calculations.

In catalytic water oxidation studies, the turnover frequency (TOF) is commonly used as a proxy for the *k_cat_* value to quantify the catalyst activity, reflecting the number of substrate molecules converted per active site per unit time. Given the inherent complexity of the four-electron oxidation mechanism, the reaction rate can be approximated via calculating the *k_cat_* (TOF). Figure 5 inset demonstrates the linear dependence of the normalized catalytic current (*i_cat_*/*i_p_*) for both complexes on the inverse square root of the scan rate (*v*^−1/2^, *s*^−1/2^), consistent with diffusion-controlled kinetics. The turnover frequency (TOF) of complex **1** is 7.23 s^−1^, which outperforms many reported water oxidation catalysts (as shown in Table S3). In contrast, the TOF of complex **2** is only 0.31 s^−1^. It is speculated that the hydroxyl group on the methanol-solvated deprotonated dpk·MeOH ligand in complex **1** plays a role in the four-electron transfer process and facilitates proton-coupled electron transfer [54,56,57].

**Figure 6:**
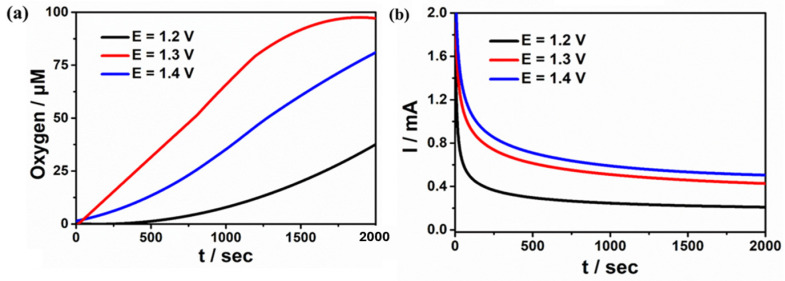
(**a**) Curves showing the variation in the dissolved oxygen concentration for complex **1** at different potentials. (**b**) Controlled potential electrolysis curves of complex **1** with a concentration of 0.2 mM on a fluorine-doped tin oxide (FTO) conducting glass working electrode with an area of 1.0 cm^2^.

Under nearly neutral conditions at pH = 8.22, we conducted controlled potential electrolysis (CPE) experiments on complexes **1** and **2** at different voltages in a sealed electrolytic cell under identical conditions. The oxygen evolution performance of complex **1** displayed voltage-dependent trends: at 1.2 V, 1.3 V, and 1.4 V, the dissolved oxygen concentrations measure d38, 97, and 81 μM (Figure 6), accompanied by current densities of 0.22, 0.42, and 0.5 mA cm^−2^, respectively. The Faraday efficiency (FE) peak is 89% at 1.3 V. For complex **2**, as shown in Figure S5, at applied potentials of 1.25 V and 1.55 V, the dissolved oxygen concentrations are 51 and 98 μM, respectively, with current densities of 0.25 and 0.41 mA cm^−2^. The Faraday efficiency (FE) peak is 92% at 1.5 V. In 0.1 M sodium acetate solution, we conducted in situ UV–Vis spectroelectrochemical monitoring on complex **1** at an applied potential of 1.3 V and complex **2** at an applied potential of 1.5 V, respectively. By utilizing controlled potential electrolysis technique, we observed the evolution of dynamic spectra (Figure S6). In particular, during the catalytic process, the absence of new peaks and the lack of significant peaks shifts indicates that no new species were formed. This demonstrates the stability of the two complexes during the catalytic process. Furthermore, comparative analysis of the scanning electron microscopy (SEM) images of complexes **1** (Figure S7a,b) and **2** (Figure S8a,b) before and after controlled potential electrolysis revealed no formation of electrode deposits, confirming their excellent structural stability under electrocatalytic conditions.

### 2.5. Study on Electrocatalytic Reduction of CO_2_ by Complexes ***1*** and ***2***

**Figure 7:**
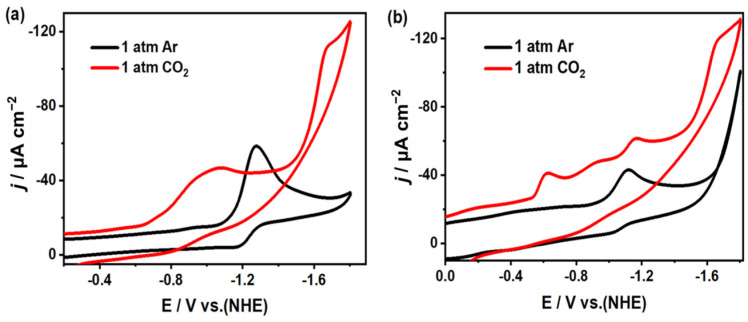
Electrochemical characterization of 0.2 mM complexes **1** (**a**) and **2** (**b**) was conducted in MeCN with 0.1 M [^n^Bu_4_N][PF_6_] as the supporting electrolyte. Comparative CV profiles under CO_2_ (red) versus Ar (black) atmospheres were recorded at 100 mV s^−1^ using a glassy carbon working electrode.

**Figure 8:**
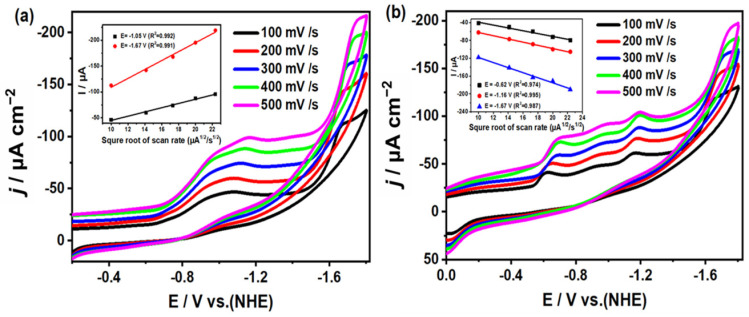
Electrochemical kinetic characteristics of 0.2 mM of complexes **1** (**a**) and **2** (**b**) were analyzed in a carbon dioxide-saturated acetonitrile (MeCN)/0.1 M tetra-n-butylammonium hexafluorophosphate ([^n^Bu_4_N][PF_6_]) electrolyte. Cyclic voltammetry graphs were recorded using a glassy carbon working electrode at various scan rates ranging from 100 to 500 mV s^−1^. The inset shows a linear relationship between the cathodic peak current density and the square root of the scan rate.

In an Ar and CO_2_-saturated acetonitrile solution (with 0.1 M ^n^Bu_4_NPF_6_ as the electrolyte), the electrocatalytic CO_2_ reduction performance of complexes **1** and **2** was evaluated using cyclic voltammetry (CV) and controlled potential electrolysis (CPE). By comparing the CV analysis under argon (Ar) and CO_2_ atmospheres, distinct differences in catalytic responses were revealed. As shown in Figure 7, compared to the measurements under an inert atmosphere, complex **1** exhibited two enhanced reduction peaks at −1.05 V and −1.67 V under CO_2_-saturated conditions. Similarly, complex **2** showed three amplified reduction peaks at -0.62 V, −1.16 V, and −1.67 V. In addition, during the CPE experiments, the products at the aforementioned potentials were analyzed, and CO produced through the electrocatalytic reduction of CO_2_ was detected using gas chromatography (GC) and ^1^H NMR (Figure S9). This further confirms that complexes **1** and **2** are capable of electrocatalyzing the reduction of CO_2_ to CO and no other liquid product. Meanwhile, by comparing the SEM images of complexes **1** (Figure S7c,d) and **2** (Figure S8c,d) before and after electrolysis, it was found that no electrodeposited films appeared, indicating that the structures of complexes **1** and **2** remained essentially unchanged during the electrolysis process. Additionally, CV experiments for complexes **1** and **2** under saturated CO_2_ in MeCN solution were performed at various scan rates, as shown in Figure 8. It was observed that all the CV curves at different scan rates exhibited good reproducibility, without the emergence of new oxidative or reductive peaks. This confirms the reproducibility and stability of the electrocatalytic CO_2_ reduction for both complexes. Meanwhile, an investigation into the relationship between the complex concentrations and the catalytic current peaks (Figure S10) revealed a linear dependence of the catalytic currents at the cathode potentials on the catalyst concentrations, indicating that the rate-determining step in the catalysis is first-order.

The turnover frequency (TOF) was determined using Equation (4) from catalytic CVs [38].

(4) $$TOF=\frac{Fvn_{p}^{3}}{RT}(\frac{0.4463}{n_{cat}}{)}^{2}(\frac{i_{cat}}{i_{p}}{)}^{2}$$

*F* is Faraday’s constant (96485 C mol^−1^), *v* is the scan rate employed (0.1 V s^−1^), *n_p_* is the number of electrons involved in the non-catalytic redox reaction (*n_p_* = 1), *R* is the gas constant (8.314 J K^−1^ mol^−1^), *T* is the temperature (293.15 K), and *n_cat_* is the number of electrons involved for the catalytic reaction (*n_cat_* = 2 for the reduction of CO_2_ to CO or the evolution of H_2_), and *i_p_* and *i_cat_* are determined as the peak current under Ar and CO_2_, respectively. The calculated TOF is 0.715 s^−1^ (*i_cat_*/*i_p_* = 1.92) in MeCN solution at the potential of −1.67 V for complex **1** and 0.4 s^−1^ (*i_cat_/i_p_* = 1.44) for complex **2**.

**Figure 9:**
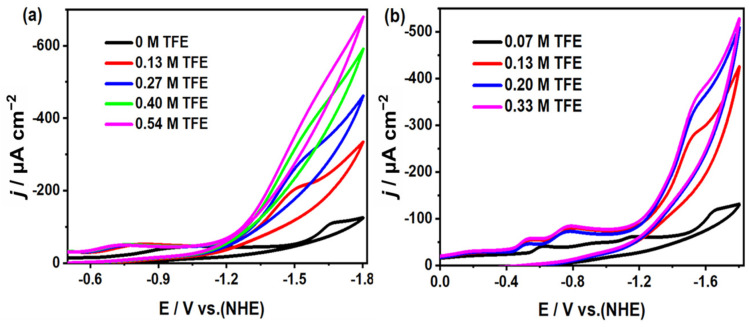
Electrochemical evaluation of 0.2 mM complexes **1** (**a**) and **2** (**b**) was conducted in CO_2_-saturated MeCN containing 0.1 M [^n^Bu_4_N][PF_6_] as supporting electrolyte, utilizing a glassy carbon working electrode at 100 mV s^−1^. Systematic CV profiling was performed with incremental trifluoroethanol (TFE) additions to probe proton source dependency.

To assess the electrocatalytic reduction activity of CO_2_ in the presence of a proton donor system, trifluoroethanol (TFE) was incrementally introduced to the catalytic system under consistent conditions (0.1 M ^n^Bu_4_NPF_6_ in MeCN solution). Notably, upon the introduction of TFE as the proton source, the catalytic currents for both complexes at −1.67 V were significantly enhanced. The TOF for complex **1** reached 4.27 s^−1^ (*i*_cat_/*i*_p_ = 4.66) under 0.27 M TFE conditions, while the TOF for complex **2** was as high as 8.9 s^−1^ (*i_cat_*/*i_p_* = 6.78) under 0.2 M TFE conditions (Figure 9). Furthermore, an increase in the TFE concentration led to a shift in the catalytic peak potentials towards the anode for both systems, which is superior to most of the reported homogeneous copper-based and transition metal-based CO_2_ reduction electrocatalysts (Table S4). These values are higher than those obtained under identical conditions without TFE, further confirming that the incorporation of a proton source enhances the catalysis and increases the TOF.

## 3. Experiments

### 3.1. General Procedures

Since oxygen can affect the redox peaks of complexes, it is imperative to conduct the electrochemistry property testing of two complexes under an argon atmosphere. Similarly, during the testing of the electrocatalytic water oxidation performance of these two complexes, the oxygen in the air can also influence the test results, necessitating the use of an argon atmosphere for this process as well. In the experiment of electrocatalytic carbon dioxide reduction, the solution needs to be saturated with carbon dioxide, meaning that the solvent for electrocatalysis is saturated with carbon dioxide. Therefore, unless specified otherwise, all procedures were performed in an argon or CO_2_ atmosphere, utilizing chemicals sourced commercially and employed directly as received, without additional purification steps. The argon and CO_2_ gases were acquired from Deihai Gases Corporation of China.

### 3.2. Synthesis

Synthesis of complex **1**: Di(2-pyridyl) ketone (1 mmol, 0.1842 g) was dissolved in 10 mL of a 1:1 methanol/acetonitrile mixture. After complete dissolution, 300 μL of triethylamine (TEA) was added, and the solution was stirred for 20 min to form a colorless transparent mixture. Subsequently, CuCl_2_·2H_2_O (1 mmol, 0.1705 g) was added, resulting in a pale green solution. The reaction mixture was stirred at room temperature under ambient air condition for 24 h and then filtered. The filtrate was subjected to vapor diffusion using diethyl ether (Et_2_O) at room temperature for approximately three days, yielding pale green block-shaped crystals. The crystals were washed with Et_2_O and dried in a desiccator. C_24_H_23_Cl_2_Cu_2_N_4_O_4.5_: calcd. C 45.36, H 3.63, N 8.91; found C 45.22, H 3.64, N 8.79.

Synthesis of complex **2**: The synthesis method for compound **2** is similar to that of compound **1**, except that Cu(NO_3_)_2_·6H_2_O was used in place of CuCl_2_·2H_2_O. C_50_H_50_Cu_4_N_10_O_16_: calcd. C 46.15, H 3.87, N 10.76; found C 47.07 H 4.32, N 9.91.

## 4. Conclusions

In this study, di(2-pyridyl) ketone was used as a ligand to synthesize two completely new copper complexes, [Cu^II^_2_L_2_Cl_2_]·0.5H_2_O (**1**) and [Cu_4_^II^L_4_(OCH_3_)_2_](NO_3_)_2_ (**2**) (L = deprotonated methoxy-di-pyridin-2-yl-methanol). The binuclear complex **1** features two Cu(II) centers with an identical coordination environment comprising two deprotonated dpk·MeOH ligands and two chloride ions, each copper atom being pentacoordinated. The tetranuclear complex **2**, on the other hand, is coordinated by four deprotonated dpk·MeOH ligands and two methoxy groups, with two copper ions in a four-coordinate state and the other two in a six-coordinate state. The significantly different geometric structures of these two complexes result in their distinct catalytic properties. Both complexes **1** and **2** demonstrate remarkable electrocatalytic performance for both water oxidation and CO_2_ reduction; nevertheless, each has its own respective advantages in terms of these two properties. Specifically, complex **1** exhibits a turnover frequency (TOF) of 7.23 s^−1^ for water oxidation and 4.27 s^−1^ for the catalytic reduction of CO_2_ to CO, while complex **2** shows a TOF of 0.31 s^−1^ for water oxidation and 8.9 s^−1^ for CO_2_ reduction. The highest TOF values of these two complexes for electrocatalytic water oxidation and carbon dioxide reduction are higher than or comparable to those reported in the literature. The binuclear complex **1** exhibits a high turnover frequency (TOF) for water oxidation, which may be attributed to its binuclear structure facilitating proton-coupled electron transfer during the process. The tetranuclear complex **2**, on the other hand, demonstrates exceptional CO_2_ reduction activity, which is likely due to its multinuclear core structure enhancing the adsorption and activation capabilities of CO_2_ molecules. Complexes **1** and **2** are pioneering examples of copper molecular electrocatalysts that possess the capability to catalyze both water oxidation and CO_2_ reduction. The dpk ligand’s redox activity and tunable coordination modes provide a blueprint for designing next-generation catalysts for energy conversion.

## Data Availability

Data is contained within the article.

## Supplementary Materials

The following supporting information can be downloaded at https://www.mdpi.com/article/10.3390/molecules30071544/s1. **Figure S1.** ^1^H NMR spectrum of dpk·MeOH ligand: (a) full spectrum and (b) partially enlarged spectrum (6.0–9.0 ppm): 3.3 ppm: methanol methyl hydrogen (CH_3_-O-); 4.9 ppm: hemiketone hydroxy hydrogen (-OH); 5.3 ppm: hemiketone hypomethylhydrogen (-CH(O-)-O-); 7.5–8.7 ppm: pyridine cycloaromatic hydrogen; **Figure S2.** Mass spectrometry results for complex **1** (a) and complex **2** (b). Complex **1**, with the chemical formula C_24_H_23_Cl_2_Cu_2_N_4_O_4.5_ (molecular weight 637.47), after deducting the crystalline water (0.5H_2_O ≈ 9 Da) and adding a hydrogen ion (H^+^), has a theoretical value for the molecular ion peak of 628.47 + 1 ≈ 629.47. The actual measured m/z of 629.49 corresponds to the main peak of complex **1** (singly charged ion). Complex **2**, with the chemical formula C_50_H_50_Cu_4_N_8_O_10_ (molecular weight 1177.14), has a theoretical m/z for the molecular ion peak of 1177.14 / 2 ≈ 588.57. The actual measured m/z of 588.37 corresponds to the main peak of complex **2** (doubly charged ion). The slight deviations may be attributed to isotope effects and instrumental errors; **Figure S3**. Ten scanning cycles of 0.2 mmol/L complexes **1** (a) and **2** (b) MeCN under 1 atm Ar (scan rate = 100 mV/s); **Figure S4.** Cyclic voltammograms of complexes **1**(a) and **2**(b) in 0.1 M acetate buffer with a glassy carbon electrode and a scan rate of 100 mV s^−1^. (inset) Relationship between oxidation peak current and concentration; **Figure S5.** (a) The dissolved O_2_ concentration trace for complex **2** during electrolysis. (b) A CPE with 0.2 mM complex **2** on the FTO working electrode (1.0 cm^−2^); **Figure S6.** (a) UV–Vis spectroelectrochemical diagrams of complex **1** (a) under an applied potential of 1.3 V and complex **2** (b) under an applied potential of 1.5 V in 0.1 M sodium acetate solution at 1 atm Ar; **Figure S7.** Scanning electron microscopy before (a) and after 2000 s electrolysis (b) in 0.1 M acetate buffer solution under 1 atm Ar of complex **1**; scanning electron microscopy before control potential electrolysis (c) and after 3600 s electrolysis (d) in a MeCN solution containing 0.1 M [^n^Bu_4_N][PF_6_] under saturated CO_2_ of complex **1**; **Figure S8.** Scanning electron microscopy before (a) and after 2000 s electrolysis (b) in 0.1 M acetate buffer solution under 1 atm Ar of complex **2**; scanning electron microscopy before control potential electrolysis (c) and after 3600 s electrolysis (d) in a MeCN solution containing 0.1 M [^n^Bu_4_N][PF_6_] under saturated CO_2_ of complex **2**; **Figure S9.** ^1^H NMR spectra of complexes **1** and **2**; **Figure S10.** Cyclic voltammetry of 0.2 mM complexes **1**(a) and **2**(b) in a MeCN solution containing 0.1 M [^n^Bu_4_N][PF_6_] under an atmosphere of CO_2_ with a glassy carbon electrode in different concentrations (the scanning rate is 100 mV s^−1^). (inset) Relationship between complex concentration and reduction peak current; **Figure S11.** Schematic diagram of the electrocatalytic device: electrocatalytic water oxidation (a) and electrocatalytic CO_2_ reduction (b). **Table S1**. Crystallographic data and structure correction parameters of complexes **1** and **2**; **Table S2**. Main bond length and bond angle data of complexes **1** and **2**; **Table S3.** Comparison of different parameters of complexes for water oxidation in recent years [38,58,59,60]; **Table S4.** Comparison of different parameters of complexes for carbon dioxide reduction in recent years [61,62,63,64,65].
