# Supplementary material for: Electrocatalytic CO2 Reduction Coupled with Water Oxidation by bi- and Tetranuclear Copper Complexes Based on di-2-pyridyl Ketone Ligand
Source: Molecules. 2025 Mar 31;30(7):1544. doi: 10.3390/molecules30071544 (PMC11990216; doi:10.3390/molecules30071544)
Supplement: Supplementary file 1 [file molecules-30-01544-s001.zip › molecules-3468790-supplementary.pdf]

SUPPORTING INFORMATION:

Electrocatalytic CO_2 reduction coupled with water oxidation by bi- and Tetranuclear copper complexes based on di-2-pyridyl ketone ligand

Siyuan Yang†, Tian Liu†, Wenbo Huang, Chengwen Zhang and Mei Wang*

School of Materials Science and Engineering, Institute for New Energy Materials & Low Carbon Technologies, Tianjin University of Technology, Tianjin 300384 (China)

† These authors contributed equally to this work.

*** Correspondence: meiwang@email.tjut.edu.cn**

Contents **Page No**

Crystal Structure Determination 3

Electrochemical measurements and Electrolysis Product Analysis 3

Figure S1. ^{1}H NMR of dpk·MeOH: (a) full spectrum and (b) partially enlarged spectrum (6.0-9.0ppm) 4

Figure S2. Mass Spectrometry Results for complex 1 (a) and complex 2 (b) 4

Figure S3. Ten scanning cycles of 0.2 mmol/L complexes 1 (a) and 2 (b) MeCN under 1 atm Ar (scan rate = 100 mV/s). 5

Figure S4. Cyclic voltammograms of complexes 1(a) and 2(b) in 0.1 M acetate buffer with a glassy carbon electrode and a scan rate of 100 mV s^{-1}. (inset) Relationship between oxidation peak current and concentration. 6

Figure S5. (a) The dissolved O_2 concentration trace for complex 2 during electrolysis. (b) A CPE with 0.2 mM complex 2 on the FTO working electrode (1.0 cm^{-2}). 7

Figure S6. (a) UV-vis spectroelectrochemical diagrams of complex 1 (a) under an applied potential of 1.3 V and complex 2 (b) under an applied potential of 1.5 V in 0.1 M sodium acetate solution at 1 atm Ar. 8

Figure S7. Scanning electron microscopy before (a) and after 2000 s electrolysis (b) in 0.1 M acetate buffer solution under 1 atm Ar of complex 1; Scanning electron microscopy before control potential electrolysis (c) and after 3600 s electrolysis (d) in a MeCN solution containing 0.1 M [^{n}Bu$_4$N][PF$_6$] under saturated CO_2 of complex 1. 8

Figure S8. Scanning electron microscopy before (a) and after 2000 s electrolysis (b) in 0.1 M acetate buffer solution under 1 atm Ar of complex 2; Scanning electron microscopy before control potential electrolysis (c) and after 3600 s electrolysis (d) in a MeCN solution containing 0.1 M [^{n}Bu$_4$N][PF$_6$] under saturated CO_2 of complex 2. 9

Figure S9. ^{1}H NMR Spectra of complexes 1 and 2 9

Figure S10. Cyclic voltammetry of 0.2 mM complexes 1(a) and 2(b) in a MeCN solution containing 0.1 M [$^nBu_4N$][PF_6] under an atmosphere of CO_2 with a glassy carbon electrode in different concentrations (the scanning rate is 100 mV s^{-1}). (inset) Relationship between complex concentration and reduction peak current. 10

Figure S11. Schematic diagram of the electrocatalytic device. 11

Table S1. Crystallographic data and structure correction parameters of complexes 1 and 2.....12

Table S2. Main bond length and bond angle data of complexes 1 and 2. 13

Table S3. Comparison of different parameters of complexes for water oxidation in recent years. 14

Table S4. Comparison of different parameters of complexes for carbon dioxide reduction in recent years...14

Crystal Structure Determination

Single crystal of **1** and **2** were mounted on a Bruker SMART APEX II CCD X-ray single-crystal diffractometer from Bruker, Germany, and all data were collected at 173 K with graphite monochromated MoKα radiation (λ = 0.71073 Å) in I > 2σ(I) diffraction spots and reduced by the SAINT program, and absorption corrections were applied using program SADABS [1]. Due to the significant disorder in the molecule's anion of complex **2**, the Platon-Squeeze method was employed to refine the structure of complex **2**, excluding the disordered anion. The CCDC numbers for complexes **1** and **2** are 2007671 and 2007670, respectively [2].

Electrochemical measurements and Electrolysis Product Analysis

All electrochemical experiments were conducted using a CHI660E electrochemical analyzer, which is manufactured by Shanghai Chenhua Instrument Co., Ltd. in China. Both cyclic voltammetry (CV) and controlled potential electrolysis (CPE) experiments were performed in a three-electrode electrochemical cell, with 0.1 M/L $^{n}Bu_4NPF_6$ in acetonitrile serving as the supporting electrolyte. The working electrode was a fluorine-doped tin oxide (FTO) conductive glass substrate (1 cm × 1 cm, with an effective surface area of 1.0 cm^2); the reference electrode was Ag/Ag; and the counter electrode was a platinum wire. The principle of the electrocatalytic device is shown in Figure S11. Prior to use, the working electrode was soaked in a 5 wt% aqueous solution of sodium hydroxide in ethanol and subsequently rinsed with water, ethanol, and water again. The specific operation involved adding 0.004 mol of the complex to 20 ml of the electrolyte solution, sonicating to dissolve, and then placing it in the electrochemical cell. Before the experiment, the solution was purged with argon or carbon dioxide for 30 minutes to eliminate air interference.

The real-time monitoring of electrochemical processes was conducted using a Shimadzu UV-1800 spectrophotometer for simultaneous spectroscopic and electrochemical analysis. The manufacturer of the Shimadzu UV-1800 UV-Visible Spectrophotometer is Shimadzu Corporation, headquartered in Kyoto, Japan. A customized three-electrode system was established in a 1 cm pathlength quartz cuvette, employing platinum gauze as the working electrode, platinum wire as the counter electrode, and a saturated KCl Ag/AgCl reference electrode to complete the electrochemical cell configuration. For CO_2 conversion product analysis, gas phase components were quantitatively determined through gas chromatography. Headspace samples (1 mL) collected during controlled potential electrolysis were injected into a Shimadzu GC-2014 system featuring dual detection capabilities: a methantor equipped flame ionization detector for CO quantification and a thermal conductivity detector for H_2 analysis, with ultra-pure argon serving as the carrier gas. The manufacturer of the Shimadzu GC-2014 Gas Chromatography System is Shimadzu Corporation, headquartered in Kyoto, Japan. In oxygen evolution studies, dissolved and headspace oxygen concentrations were monitored using a NeoFox-GT fiber-optic sensor (Ocean Optics). The manufacturer of the NeoFox-GT fiber-optic sensor is Ocean Insight (formerly Ocean

Optics), headquartered in Orlando, Florida, USA. While the system successfully detected solution-phase oxygen generation during water oxidation, post-electrolysis measurements revealed that headspace oxygen levels remained below the sensor's quantification threshold of 0.1 ppm. 1H NMR tested on AVANCE III HD 400MHz instrument. The mass spectrometry test is conducted on an LTQ-XL linear ion trap mass spectrometer from Thermo Fisher Scientific, USA, in positive ion mode.

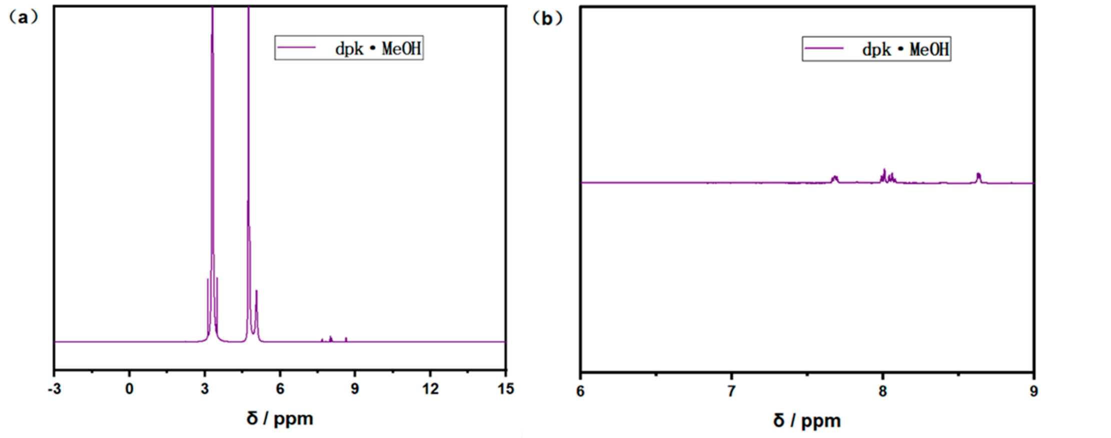

Figure S1. ^{1}H NMR spectrum of dpk·MeOH ligand: (a) full spectrum and (b) partially enlarged spectrum (6.0-9.0ppm). 3.3 ppm: methanol methyl hydrogen (CH_3-O-); 4.9 ppm: hemiketone hydroxy hydrogen (-OH); 5.3 ppm: hemiketone hypomethylhydrogen (-CH(O-)-O-); 7.5-8.7 ppm: pyridine cycloaromatic hydrogen.

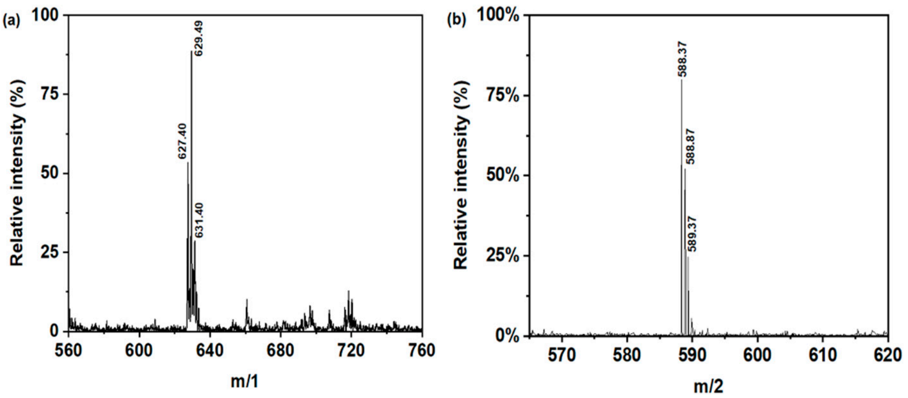

Figure S2. Mass Spectrometry Results for complex **1** (a) and complex **2** (b). Complex **1**, with the chemical formula $C_{24}H_{23}Cl_2Cu_2N_4O_{4.5}$ (molecular weight 637.47), after deducting the crystalline water ($0.5H_2O \approx 9$ Da) and adding a hydrogen ion (H^+), has a theoretical value for the molecular ion peak of 628.47 + 1 ≈ 629.47. The actually measured m/z of 629.49 corresponds to the main peak of complex **1** (singly charged ion). Complex **2**, with the chemical formula $C_{50}H_{50}Cu_4N_8O_{10}$ (molecular weight 1177.14), has a theoretical m/z for the molecular ion peak of 1177.14 /

2 ≈ 588.57. The actually measured m/z of 588.37 corresponds to the main peak of complex **2** (doubly charged ion). The slight deviations may be attributed to isotope effects and instrumental errors.

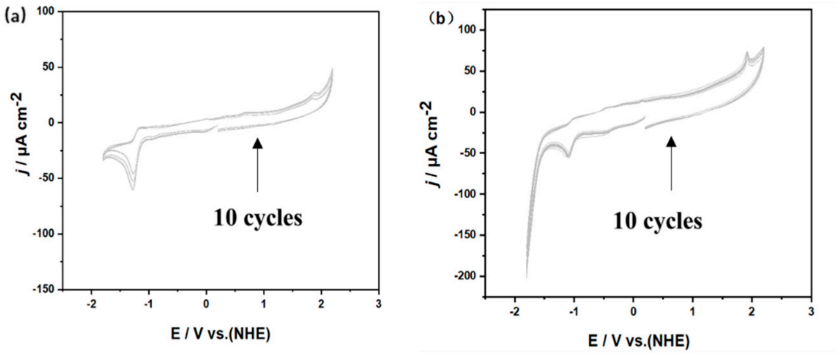

Figure S3. Ten scanning cycles of 0.2 mmol/L complexes **1** (a) and **2** (b) MeCN under 1 atm Ar (scan rate = 100 mV/s).

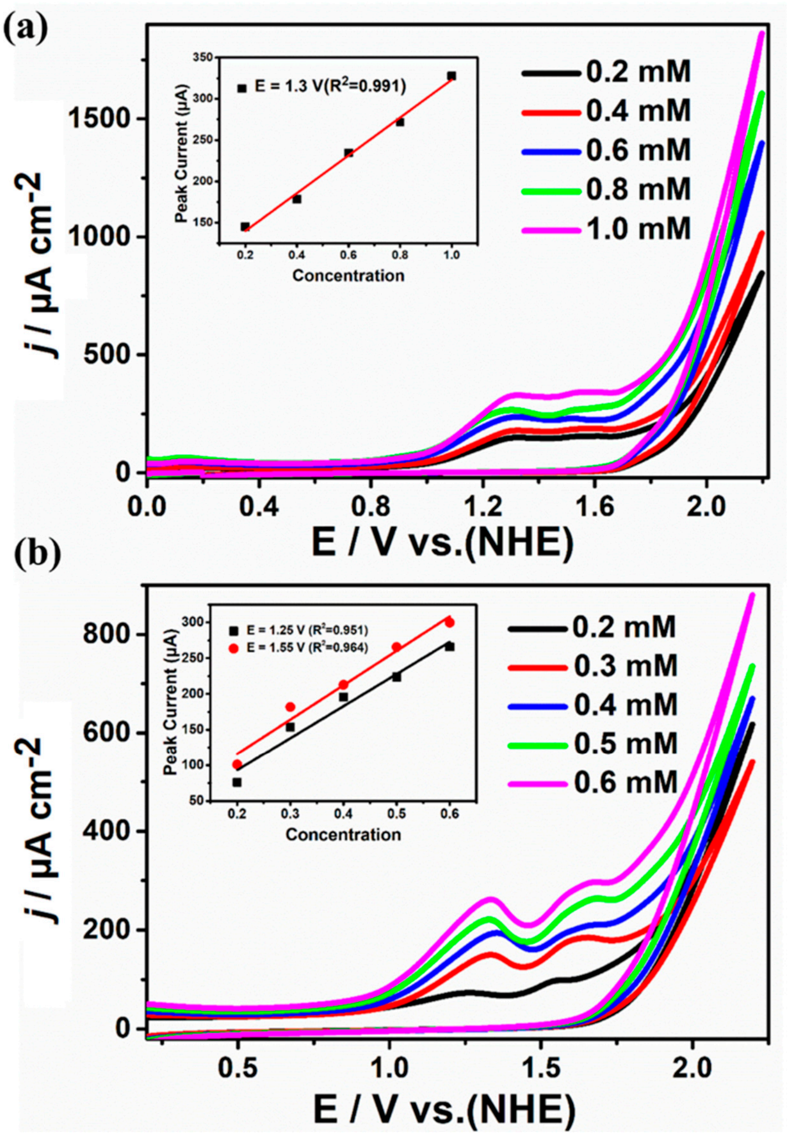

Figure S4. Cyclic voltammograms of complexes **1**(a) and **2**(b) in 0.1 M acetate buffer with a glassy carbon electrode and a scan rate of 100 mV s^{-1}. (inset) Relationship between oxidation peak current and concentration.

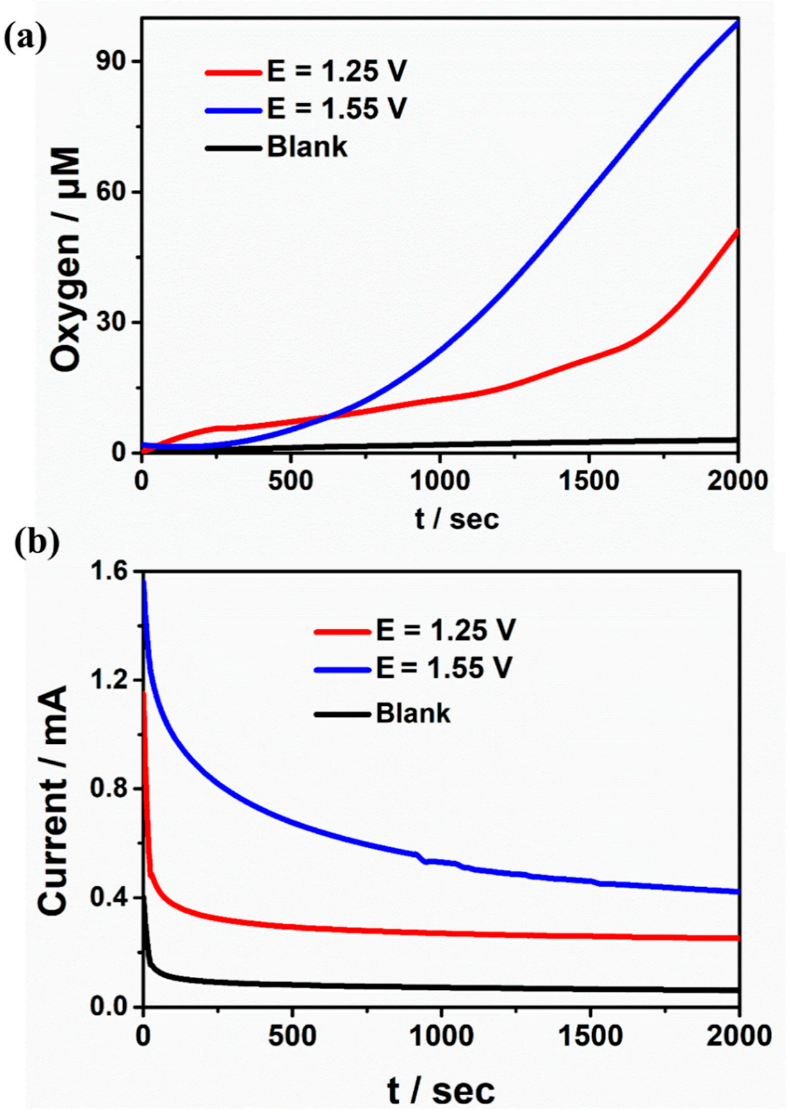

Figure S5. (a) The dissolved O_2 concentration trace for complex **2** during electrolysis. (b) A CPE with 0.2 mM complex **2** on the FTO working electrode (1.0 cm^{-2}).

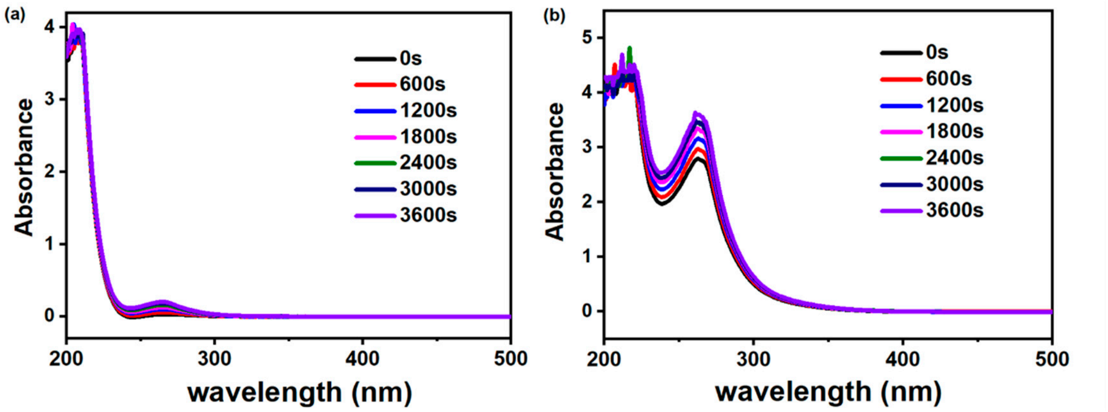

Figure S6. (a) UV-vis spectroelectrochemical diagrams of complex **1** (a) under an applied potential of 1.3 V and complex **2** (b) under an applied potential of 1.5 V in 0.1 M sodium acetate solution at 1 atm Ar.

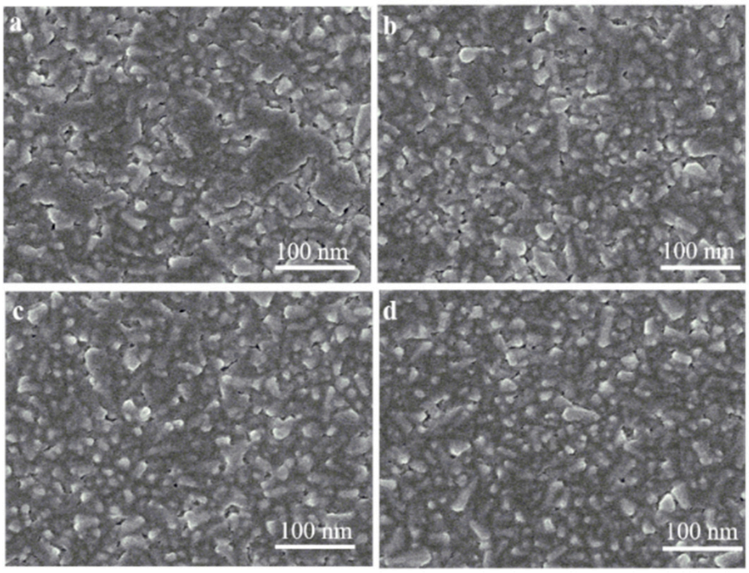

Figure S7. Scanning electron microscopy before (a) and after 2000 s electrolysis (b) in 0.1 M acetate buffer solution under 1 atm Ar of complex **1**; Scanning electron microscopy before control potential electrolysis (c) and after 3600 s electrolysis (d) in a MeCN solution containing 0.1 M [$^{n}Bu_4N$][PF_6] under saturated CO_2 of complex **1**.

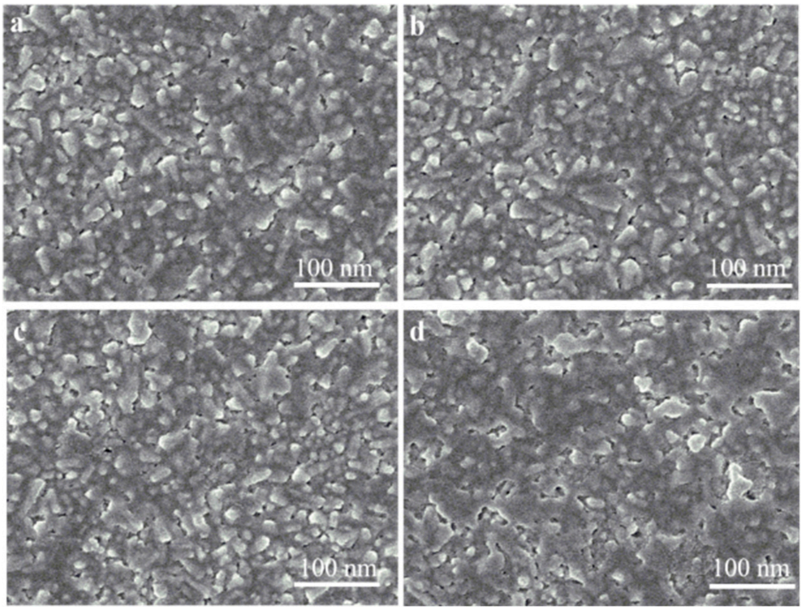

Figure S8. Scanning electron microscopy before (a) and after 2000 s electrolysis (b) in 0.1 M acetate buffer solution under 1 atm Ar of complex **2**; Scanning electron microscopy before control potential electrolysis (c) and after 3600 s electrolysis (d) in a MeCN solution containing 0.1 M [$^nBu_4N$][PF_6] under saturated CO_2 of complex **2**.

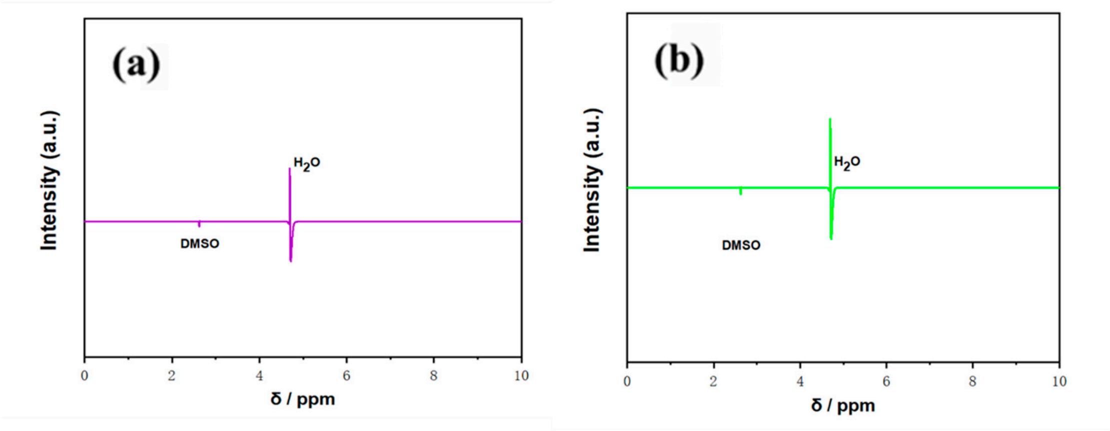

Figure S9. ^1H NMR Spectra of complexes **1** and **2**

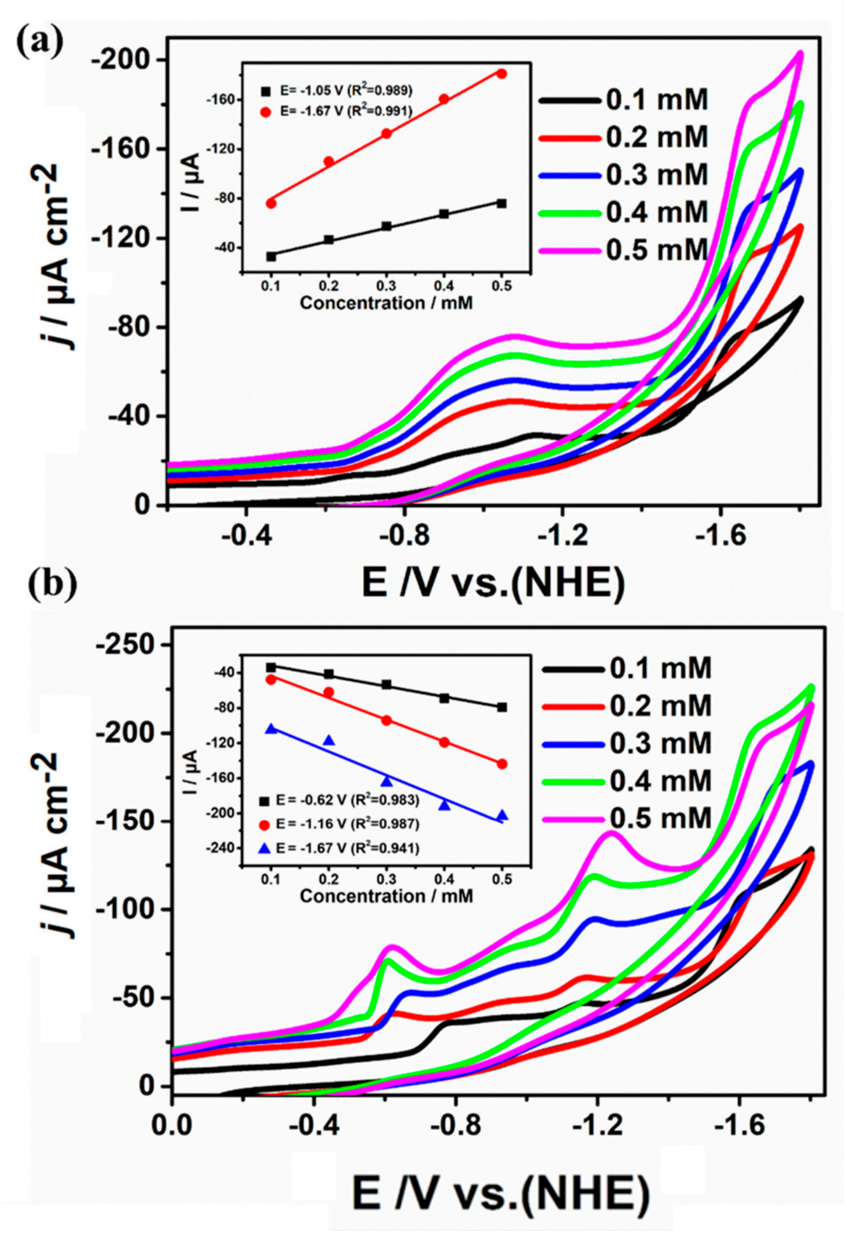

Figure S10. Cyclic voltammetry of 0.2 mM complexes **1**(a) and **2**(b) in a MeCN solution containing 0.1 M [$^nBu_4N$][PF_6] under an atmosphere of CO_2 with a glassy carbon electrode in different concentrations (the scanning rate is 100 mV s^{-1}). (inset) Relationship between complex concentration and reduction peak current.

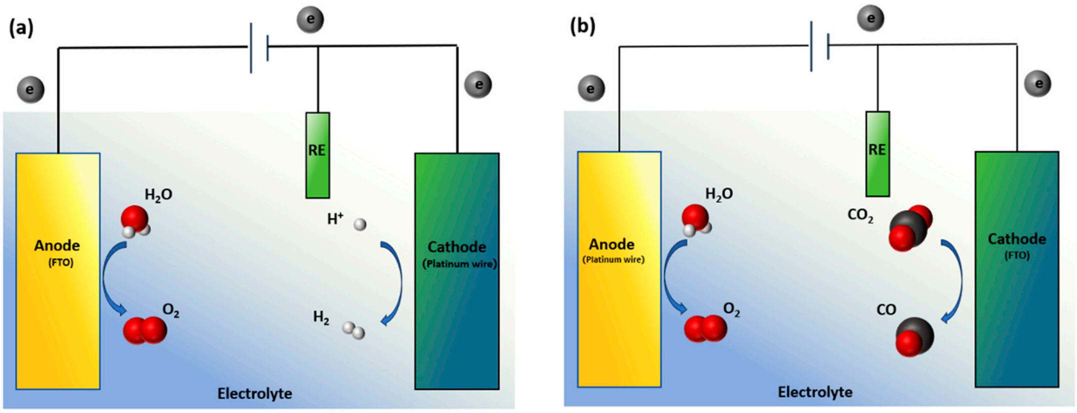

Figure S11. Schematic diagram of the electrocatalytic device: Electrocatalytic water oxidation (a) and electrocatalytic CO_2 reduction (b).

Table S1. Crystallographic data and structure correction parameters of complexes **1** and **2**.

Complex	**1**	**2**
Chemical formula	$C_{24}H_{23}Cl_2Cu_2N_4O_{4.5}$	$C_{50}H_{50}Cu_4N_8O_{10}$
Formula weight	637.47	1177.14
Crystal system	Monoclinic	Tetragonal
space group	P_{21}/n	I_{41}/acd
a (Å)	13.4639(4)	15.4876(6)
b (Å)	11.9984(3)	15.4876(6)
c (Å)	16.1287(4)	46.262(3)
α (°)	90	90
β (°)	92.7430(10)	90
γ (°)	90	90
V (Å^3)	2602.53(12)	11096.7(11)
Z	4	16
ρ_{calcd} (g m^{-3})	1.627	1.409
μ (mm^{-1})	1.880	4217
Reflections collected	9970	4217
F(000)	1296.509	4816
R_{int}	0.0393	0.0813
T (K)	170	170
Final R indices	R_1 = 0.0314	R_1 = 0.0472
[*I* > 2sigma(I)]	wR_1 = 0.0654	wR_1 = 0.1107
R indices (all data)	R_2 = 0.0460	R_2 = 0.0869
	wR_2 = 0.0743	wR_2 = 0.1283
Gof	1.0543	1.045

Table S2. Main bond length and bond angle data of complexes **1** and **2**.

Selected bond lengths (Å) of complex **1**			
Bond	Lengths (Å)	Bond	Lengths (Å)
Cu2-Cl2	2.3317(7)	Cu1-Cl1	2.2715(7)
Cu2-O4	2.0520(17)	Cu1-O4	1.9463(17)
Cu2-O3	1.9441(17)	Cu1-O3	2.0144(17)
Cu2-N3	1.980(2)	Cu1-N1	1.981(2)
Cu2-N2	2.066(2)	Cu1-N4	2.178(2)
Selected bond lengths (Å) of complex **2**			
Cu1-O1	1.972(2)	Cu1-N1	2.036(4)
Cu1-O1	1.973(2)	Cu2-O2	1.900(3)
Cu1-O2	2.399(3)	Cu2-O2	1.900(3)
Cu1-O2	2.399(3)	Cu2-N2	1.961(4)
Cu1-N1	2.036(4)	Cu2-N2	1.961(4)
Selected bond angles (deg) of complex **1**			
Bond	Angle (°)	Bond	Angle (°)
O4-Cu2-Cl3	119.21(6)	O4-Cu1-Cl1	99.59(5)
O4-Cu2-N2	124.84(8)	O4-Cu1-O3	82.75(7)
O3-Cu2-Cl2	103.87(6)	O4-Cu1-N1	160.22(8)
O3-Cu2-O4	81.83(7)	O4-Cu1-N4	78.86(7)
O3-Cu2-N3	159.50(8)	O4-Cu1-Cl1	99.59(5)
O3-Cu2-N2	80.92(8)	O3-Cu1-Cl1	141.28(5)
N3-Cu2-Cl2	94.41(7)	O3-Cu1-N4	110.28(8)
N3-Cu2-O4	81.17(8)	N1-Cu1-Cl1	100.12(7)
N3-Cu2-N2	99.60(9)	N1-Cu1-O3	80.90(8)
N2-Cu2-Cl2	115.72(7)	N1-Cu1-N4	96.47(8)
N4-Cu1-Cl1	108.05(6)		
Selected bond angles (deg) of complex **2**			
O1-Cu1-O2	74.53(18)	N1-Cu1-O2	95.27(13)
O1-Cu1-O2	96.68(7)	N1-Cu1-O2	74.09(13)
O1-Cu1-O2	94.98(8)	N1-Cu1-N1	88.9(2)
O1-Cu1-O2	96.68(7)	O2-Cu2-O2	162.18(19)
O1-Cu1-O2	94.98(8)	O2-Cu2-N2	99.72(15)
O1-Cu1-N1	99.01(13)	O2-Cu2-N2	84.38(15)
O1-Cu1-N1	168.47(12)	O2-Cu2-N2	84.38(15)
O1-Cu1-N1	168.47(12)	O2-Cu2-N2	99.73(15)
O1-Cu1-N1	99.01(13)	N2-Cu2-N2	153.5(2)
O2-Cu1-O2	165.33(15)	N1-Cu1-O2	74.09(13)
N1-Cu1-O2	95.27(13)		

Table S3. Comparison of different parameters of complexes for water oxidation in recent years.

catalyst	TOF(s-1)	ref
6-FP-Co-OMC-1	0.53	[1]
$[Fe(HbpH)(Cl)_2]$	5.8	[2]
$[Re^V(O)(Cl)_3(L^{2-})Re^V(O)(Cl)_3][NBu_4]_2$	2.1	[3]
Ni-15 film	1.01	[4]
$[Cu^{II}_2L_2Cl_2]\cdot 0.5H_2O$	7.23	This work

Table S4. Comparison of different parameters of complexes for carbon dioxide reduction in recent years.

catalyst	TOF(s-1)	ref
CNCbl-MWCNT/CP	8.2	[5]
$N_2PFe\text{-}FePN_2$	0.18	[6]
$(Et_3NH)_2[CoIII_2CoII(OH_2)(pda)_5]\cdot H_2O$	0.93	[7]
$Cr(^{tbu}dhbpy)Cl(H_2O)$	5.7	[8]
$[Ni(qlca)Cl_2]$	0.83	[9]
$[Cu_4^{II}L_4(OCH_3)_2](NO_3)_2$	8.9	This work

References

1. Makhado, T.; Das, B.; Kriek, R. J.; Vosloo, H. C. M.; Swarts, A. J. Chemical and electrochemical water oxidation mediated by bis(pyrazol-1-ylmethyl) pyridine-ligated Cu(I) complexes. *Sustain. Energ. Fuels* **2021**, *5*, 2771-2780.
2. Binaeizadeh, M.R.; Amiri, A.; Shayesteh, A.; Fadaei-Tirani, F. Carboxamide Fe (III) complex as the electrocatalyst of water oxidation reaction: WNA and I2M O–O bond formation pathways. *Int. J. Hydrog. Energy* **2024**, *51*, 709-721.
3. Shee, U.; Sinha, D.; Mondal, S.; Rajak, K.K. Electrochemical water oxidation reaction by dinuclear Re (v) oxo complexes with a 1, 4-benzoquinone core via the redox induced electron transfer (RIET) process. *Dalton Trans.* **2024**, *53*, 8254-8263.
4. Kamlesh; Aggarwal, P.; Mudgal, M.; Srivastava, A.K.; Raizada, P.; Singh, P.; Paul, A.; Singh, A. Electrochemistry of Nickelocene-Ferrocene Organometallic Complexes for Electrodeposition of Nickel–Iron–Based Nanostructured Film under Ambient Conditions for Oxygen Evolution Reaction. *ACS Appl. Nano Mater.* **2024**, *7*, 24455-24468.
5. Chen, X.; Sun, W.; Meng, X.; Gao, Y. Assembly of a Highly Efficient Molecular Device with (CNCbl)-MWCNT/CP as Electrode for CO_2 Reduction Coupled to Water Oxidation. *ChemElectroChem* **2021**, *8*, 3567-3571.
6. Guo, C.; Gao, S.; Li, J.; Zhou, M.; Abdukayum, A.; Kong, Q.; Zhou, Y.; Hu, G. P-tuned FeN_2 binuclear sites for boosted CO_2 electro-reduction. *J. Energy Chem.* **2025**, *101*, 816-824.
7. Yin, X.M.; Zhang, S.F.; Wang, J.M; Li, J.J.; Chen, F.F.; Yao, S.; Fan, Y.H.; Wang, M. Bioinspired cobalt molecular electrocatalyst for water oxidation coupled with carbon dioxide reduction. Appl. *Organomet. Chem.* **2021**, *35*, 6371.
8. Hooe, S.L.; Dressel, J.M.; Dickie, D.A.; Machan, C.W. Highly Efficient Electrocatalytic Reduction of CO_2 to CO by a Molecular Chromium Complex. *ACS Catal.* **2020**, *10*, 1146-1151.
9. Ahsan, H.Md.; Breedlove, B.K.; Piangrawee, S.; Mian, M.R.; Fetoh, A.; Cosquer, G.; Yamashita, M. Enhancement of electrocatalytic abilities for reducing carbon dioxide: functionalization with a redox-active ligand-coordinated metal complex. *Dalton. T.* **2018**, *47*, 11313-11316.
